# COVID-19 Outbreak and BNT162b2 mRNA Vaccination Coverage in a Correctional Facility during Circulation of the SARS-CoV-2 Omicron BA.1 Variant in Italy

**DOI:** 10.3390/vaccines10071137

**Published:** 2022-07-17

**Authors:** Angela Stufano, Nicola Buonvino, Claudia Maria Trombetta, Daniela Pontrelli, Serena Marchi, Giuseppe Lobefaro, Leonarda De Benedictis, Eleonora Lorusso, Maria Teresa Carofiglio, Violetta Iris Vasinioti, Emanuele Montomoli, Nicola Decaro, Piero Lovreglio

**Affiliations:** 1 Section of Occupational Medicine, Interdisciplinary Department of Medicine, University of Bari, 70124 Bari, Italy; giuseppe.lobefaro@uniba.it (G.L.); leonarda.debenedictis@uniba.it (L.D.B.); piero.lovreglio@uniba.it (P.L.); 2 U.O.C. Penitentiary Medicine, Department of Territorial Care, Bari Local Health Authority, 70124 Bari, Italy; nicola.buonvino@asl.bari.it (N.B.); daniela.pontrelli@asl.bari.it (D.P.); mariateresa.carofiglio@asl.bari.it (M.T.C.); 3 Department of Molecular and Developmental Medicine, University of Siena, 53100 Siena, Italy; trombetta@unisi.it (C.M.T.); serena.marchi2@unisi.it (S.M.); emanuele.montomoli@unisi.it (E.M.); 4 Department of Veterinary Medicine, University of Bari, 70121 Bari, Italy; eleonora.lorusso@uniba.it (E.L.); violeta.vasinioti@uniba.it (V.I.V.); nicola.decaro@uniba.it (N.D.); 5 VisMederi srl, 53100 Siena, Italy

**Keywords:** Omicron variant, prisons, inmates, correctional facilities, neutralizing antibodies, BNT162b2 vaccine, booster dose, vaccination campaign

## Abstract

**Background**. The recent spread of the highly mutated SARS-CoV-2 Omicron variant (B.1.1.529) has raised concerns about protection against COVID-19 in congregate settings such as prisons, characterized by a high risk of transmission and possible difficulties in obtaining adequate vaccination coverage. The present study aims to investigate the spread of an outbreak of COVID-19 in an Italian correctional facility during the dominant circulation of the Omicron BA.1 variant, and also considers BNT162b2 mRNA vaccination coverage among inmates. A COVID-19 screening campaign by RT-PCR was performed on 515 detainees from 4–30 January 2022, in response to an outbreak that began in the correctional facility. Furthermore, 101 serum samples collected from healthy inmates 21 days after having received the second dose of the BNT162b2 vaccine were tested for neutralizing antibodies against both the wild-type SARS-CoV-2 strain and the Omicron BA.1 variant. The global attack rate during the study period was 43.6% (RR 0.8), progressively reducing from unvaccinated inmates (62.7%, RR 1.8) to those who had one dose (52.3%, RR 1.5), two doses (full cycle) (45.0%, RR 1.3), and the third dose (booster) vaccinated group (31.4%, RR 0.7). The percentage of SARS-CoV-2 positive subjects among unvaccinated inmates was significantly higher than in the other groups (*p* < 0.001), while no significant difference was observed between inmates with one or two vaccine doses. Only two of the positive inmates were hospitalized for COVID-19. The geometric mean titer of neutralizing antibodies in the tested sub-group after two doses of vaccine was lower than in previous studies against the wild-type virus, and showed a complete lack of neutralization against the Omicron variant in 92.1% of individuals. The findings support the need to prioritize vaccination in correctional facilities, as a public health measure to increase the protection of inmates and consequently of prison workers and the community against COVID-19, in coordination with the other prevention strategies.

## 1. Introduction

Since the first reports of Coronavirus Disease 2019 (COVID-19) in humans in December 2019, several genetically distinct lineages of the etiological agent Severe Acute Respiratory Syndrome Coronavirus 2 (SARS-CoV-2) have emerged [1]. The highly mutated B.1.1.529 variant (Omicron) was firstly reported in Botswana in November 2021, and was quickly designated as a novel variant of concern (VOC) by the World Health Organization (WHO), based on higher infectiveness and possible immune evasion [2]. In fact, the Omicron variant spread rapidly across Europe, with an estimated average basic reproductive number (R0) of 9.5, much higher than that estimated for the delta variant [3]. These characteristics, along with a milder severity of clinical course, have completely changed the pandemic scenario, leading to inevitable consequences particularly within the prison system.

Correctional and detention settings are congregate living and working environments at higher risk of SARS-CoV-2 spread, due to overcrowding, poor air ventilation and less healthcare provision, facing incredible challenges in mitigating COVID-19 [4,5]. In fact, since the beginning of the pandemic, despite preventive measures and screening campaigns, incarcerated populations have experienced disproportionately higher rates of COVID-19 cases and deaths compared with the general population, also due to a high prevalence of underlying medical conditions associated with severe COVID-19 [6,7]. Moreover, prison should be considered as a reservoir of infection for the community, considering that it is an open dynamic system, due to inmate turnover, the presence of workers performing different jobs (correctional officers, health care and administrative workers), and relationships with external visitors (e.g., family members, lawyers, etc.) [8]. Failure to control infection in prisons, therefore, poses a high risk to the general population. 

Vaccination has played an effective role in COVID-19 prevention, reducing incidence, hospitalization, and mortality. BNT162b2 (Pfizer-BioNTech, Mainz, Germany) was the first vaccine to be granted temporary authorization for emergency use in Italy in December 2020, followed by 1273 (Moderna, Cambridge, MA, USA) in December 2020, both developed using the mRNA vaccine platform. Three other vaccines have been authorized in Italy, two adenovirus vector vaccines, the recombinant adenoviral AZD1222 or ChAdOx1-S (Oxford/AstraZeneca, Cambridge, UK), Ad26.COV2S (Janssen/Johnson & Johnson, Beerse, Belgium), and NVX-CoV2373 (Novavax, Gaithersburg, MD, USA) which is a recombinant SARS-CoV-2 nanoparticle vaccine [9]. However, all these authorized vaccines use the SARS-CoV-2 spike (S) protein of the ancestral wild-type (WT) strain as a template to induce immune responses [10], while the large number of mutations and deletions in the Omicron S protein could have reduced the effectiveness of vaccine protection [11].

Although incarcerated people have not been included in vaccine trial populations, vaccination could represent a key tool of the multicomponent strategy to prevent COVID-19 in prisons [12,13]. For this reason, most scientists and public health experts have recommended prioritization of inmates and prison and jail workers for deployment of COVID-19 vaccines. However, limited information is available on the effectiveness of vaccine and breakthrough infections in correctional and detention settings after the emergence and spreading of Omicron, partly because vaccine rollout has varied across prisons in different countries, despite the scientific recommendations [14]. In this context, neutralizing antibodies seem to be strongly predictive of the degree of immune protection against SARS-CoV-2 infection, and their determination can improve knowledge of the real immune escape due to new mutations in the Omicron variant infecting vaccinated individuals [15].

The aim of this study was to evaluate how the vaccination coverage by the BNT162b2 vaccine in a correctional facility influenced an outbreak of COVID-19 among the inmates during the Omicron BA.1 wave of the pandemic. We also investigated the neutralizing antibodies response in a sub-group of healthy prisoners after the administration of two doses of the mRNA vaccine.

## 2. Materials and Methods

### 2.1. Correctional Facilities

The study was performed at the Bari (Apulia, South Italy) correctional facility, and at two further smaller sites (Altamura and Turi), located at an average distance of about 40 km, belonging to a single central management.

As previously described, Bari prison (site 1) is a penitentiary where most of the residential inmates are treated for chronic diseases, and some suffer from severe disabilities [8]. At the time of the survey, it had a capacity of 299 male residential inmates, with an average occupancy rate of 153%. The Altamura prison (site 2) is made up of three sections, including one section dedicated to semi-free prisoners. At the time of the study, there were 80 male inmates. Inside the institute there was a system of dynamic surveillance, allowing inmates to spend more than 12 h a day outside their cells and even to leave their section. The Turi prison (site 3) housed 112 male inmates, all with final sentences, with a regulatory capacity of 108 people. All the three penitentiaries applied the same COVID-19 prevention protocols previously described [8]. 

### 2.2. SARS-CoV-2 Vaccination Campaign

**Figure 1:**
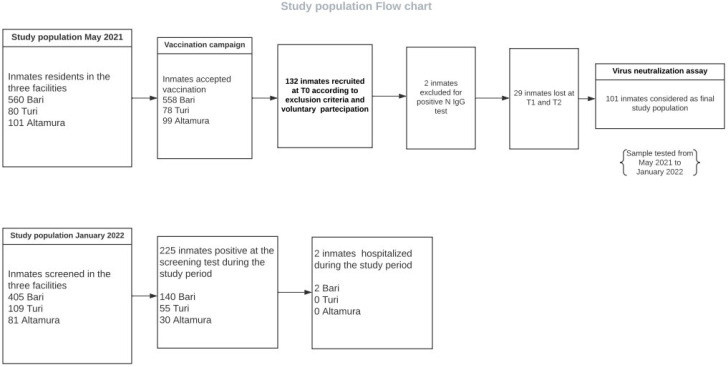
Study population flow chart.

Starting from April 2021, the BNT162b2 vaccine against SARS-CoV-2 was offered to all the eligible population housed in the three sites and the employees working there (Figure 1). To support the vaccination campaign, public health educators and medical correctional staff provided education on the vaccine, answered questions, and obtained consent before the vaccine clinic day with the option to accept or defer. By May 2021, the entire correctional population had received at last one opportunity for starting the vaccination cycle. Second-dose vaccine administration was provided at appropriate time intervals (21 days after the first dose). After the completion of these phases, the vaccine continued to be offered upon request, and by November 2021 booster doses had been offered to all the subjects who had completed the vaccination cycle. All the individuals received the BNT162b2 vaccine for all the doses.

### 2.3. COVID-19 Outbreak and Screening Campaign

The screening campaign was performed at all three sites from 4th–30th January 2022 in response to an outbreak that occurred in the first week of January 2022 in the Bari facility. According to the definition by US Center for Diseases Control (CDC), an outbreak was considered to involve more than one resident or staff member diagnosed as a SARS-CoV-2 confirmed case through antigenic or RT-PCR testing [16]. All prisoners were required to receive RT-PCR testing every 7 days until no new cases occurred for at least 14 days (Figure 1). Moreover, RT-PCR testing was offered to subjects with symptoms suggestive of COVID-19, regardless of the screening timing. All the tests were performed on a voluntary basis, after written informed consent had been obtained. Procedures for swab sample collection and for the management of positive subjects and close contacts were described in our previous study [8].

COVID-19 incidence during the investigation period was assessed across four groups, based on SARS-CoV-2 vaccination status at the time the positive respiratory specimen was collected:

(1) Unvaccinated: never received a vaccine dose.
(2) Partially vaccinated: received the first dose of the two-dose series.
(3) Fully vaccinated: received the second dose of the two-dose series.
(4) Boostered: received a third dose after the two-dose series.

Inmates receiving the last dose of vaccine in the 14 days before the test were excluded from the subsequent analysis.

The real-time RT-PCR assay for the screening was performed in a laboratory accredited by the Local Health Authority, targeting SARS-CoV-2 envelope (E), replicase (RdRP), and nucleocapsid (N) genes, according to the WHO protocol [17]. Briefly, nasopharyngeal and oronasal swabs were subjected to nucleic acid extraction using the MagNA Pure System (Roche Diagnostics, Basel, Switzerland), in accordance with the manufacturer’s instructions. The presence of the SARS-CoV-2 genes was evaluated by a commercial real-time RT-PCR assay (Allplex 2019-nCoV Assay; Seegene, Seoul, Korea). Amplification and detection were performed for 45 cycles on a BioRad CFX96 thermocycler (BioRad Laboratories, Lunteren, The Netherlands). Samples were considered positive at molecular screening if all three E, N and RdRP genes were detected.

### 2.4. Humoral Immunity Survey: Study Cohort and Sample Collection

This part of the study was performed on healthy prisoners at the Altamura and Turi penitentiary sites, recruited on a voluntary basis during the first cycle of the vaccination campaign (April–May 2021), to verify the humoral immunity response in fully vaccinated subjects not previously affected by COVID-19. Therefore, the exclusion criteria for the enrollment were a history of debilitating chronic diseases requiring hospitalization, conditions of immune-depression, and a previous history of infection by SARS-CoV-2. 

Serum samples were collected by adequately trained medical staff at three different stages: the day before administration of the first dose of the BNT162b2 vaccine (T0), the day before administration of the second vaccine dose (21 days after the administration of the first dose) (T1), and 21 days after the administration of the second vaccine dose (T2). 

Out of 132 inmates recruited according to the exclusion criteria, collection of serum samples at all three stages was only possible for 103, while the determination of IgG against N protein antibodies at T0 allowed the identification and exclusion of two more subjects with previous SARS-CoV-2 infection in the absence of anamnestic evidence (Figure 1). Assuming a final population of 101 inmates, T1 and T2 samples were divided into different aliquots used for the determination of IgG against viral protein S (T2 only), and to assess the titers of neutralizing antibodies against WT strain by the virus neutralization (VN) assay. In the period January–February 2022, during the outbreak previously described, these T1 and T2 aliquots were furtherly analyzed by VN assay to assess the titers of neutralizing antibodies against the Omicron variant.

The research protocol was approved by the Ethics Committee of the University Hospital of Bari (n. 6955, prot. N. 0067544–02082021). The serum survey was conducted in accordance with ethical principles (Declaration of Helsinki), and written informed consent was obtained from all the participants. 

#### 2.4.1. Enzyme-Linked Immunosorbent Assay (ELISA)

A double antigen ELISA kit was used for detection of antibodies against the SARS-CoV-2 N protein in human serum samples. IgG antibodies binding to SARS-CoV-2 N protein were determined using the ERADIKIT COVID19- MULTISPECIES (In3diagnostic, Turin, Italy). The results were defined based on the calculated ratio described in the following formula, and expressed as a percentage: PR (%) = (OD test sample − OD negative control)/(OD positive control − OD negative control). Values ≥ 20% were considered positive for the presence of antibodies against SARS-CoV-2. IgG antibodies binding to the SARS-CoV-2 S protein were determined using the IDK anti-SARS-CoV-2 IgG ELISA kit (Immundiagnostik AG, Bensheim, Germany). The results were defined using a linear ordinate for optical density and a logarithmic abscissa for concentration. The obtained numbers were multiplied by the dilution factor of 101 to get the actual concentrations in ng/mL. Values ≥ 175 ng/mL were considered positive for the presence of antibodies against SARS-CoV-2.

#### 2.4.2. Virus Neutralization Assay

To assess the production of neutralizing antibodies, authentic WT SARS-CoV-2 virus, 2019-nCov/Italy-INMI1 strain, was purchased from the European Virus Archive Global (EVAg, Spallanzani Institute, Rome, Italy), while the Omicron variant (sublineage BA.1) was kindly provided by Prof. Piet Maes, NRC UZ/KU Leuven (Leuven, Belgium). The Omicron sequence was deposited on GISAID with the following ID: EPI_ISL_6794907.

The VN assay was performed as previously described [18]. Serum samples were heat-inactivated for 30 min at 56 °C, two-fold serially diluted (starting from dilution 1:10), and then mixed with an equal volume of SARS-CoV-2 viral suspension containing 100 tissue culture infectious dose 50% (TCID50). After 1 h of incubation at room temperature, 100 µL of each virus-serum mixture was added to a 96-well plate containing an 80% confluent Vero E6 cell monolayer. Plates were incubated at 37 °C for 72 h for WT strain and 96 h for Omicron, in a humidified atmosphere with 5% CO_2_, then inspected for the presence or absence of cytopathic effect (CPE) by means of an inverted optical microscope. A CPE higher than 50% indicated infection. The highest serum dilution that protected more than the 50% of cells from CPE was expressed as the neutralization titre. VN titer was expressed as the geometric mean titers (GMT) of the two replicates, and GMT lower than 5 was considered negative.

### 2.5. Statistical Analysis

Statistical analysis was performed using the SPSS program (version 14, Chicago, IL, USA). For descriptive statistics, continuous variables were expressed as geometric mean and range, and non-continuous variables as frequencies and percentages. Parametric andnon-parametric tests were used for the analysis. Statistical significance was set at *p* < 0.05, adjusted for multiple comparisons using Bonferroni correction method. The receiver operating characteristic (ROC) curve was used to evaluate the predictive value of T2 anti-S IgG titers, associated with protective neutralizing antibodies GMT for WT strain and Omicron, which was defined as higher than 19 GMT according to the level reported by Khoury et al. [15] in subjects fully vaccinated by the BNT162b2 vaccine.

## 3. Results

The 595 inmates tested in all the three prisons during the study period showed a median age of 40.0 years (range 18–81 years), with a significant (*p* < 0.001) higher percentage of subjects aged 30–39 (30.0%) and 40–49 years (26.2%) (Table 1). The median number of days since the last vaccine dose was 27.5 days (range 2–282 days), with 16.0% having received their last dose of vaccine in the 14 days prior to testing. At the time of the outbreak, 84.2% of inmates reported receiving at least one dose of vaccine, with a significantly higher percentage of subjects having received a booster dose (48.9%; *p* < 0.001), while 15.8% were unvaccinated, 27.4% fully vaccinated, and 7.9% partially vaccinated (Table 1). 

The global attack rate during the study period, excluding subjects who had received their last dose of vaccine in the 14 days prior to testing, was 43.6% (RR 0.8), progressively diminishing from unvaccinated (62.7%, RR 1.8) to boostered (31.4%, RR 0.7) inmates (Table 2). The percentage of SARS-CoV-2 positive subjects among unvaccinated inmates was significantly higher than in the other groups (*p* < 0.001), while no significant difference was observed between inmates with at least one (52.3%, RR 1.5) or two doses (45.0%, RR 1.3) of vaccine (Table 2). Moreover, the boostered inmates’ attack rate was considerably lower compared with the other groups (*p* < 0.001). An attack rate of 28.6% was observed among inmates with 15–40 days since their last dose of vaccine, lower than among inmates receiving the last dose of vaccine more than 41 days prior testing. Mean number of days since last vaccine dose was significantly higher in subjects with a positive test for COVID-19 (82.7 versus 60.2 days, *p* < 0.001). Finally, no significant difference was found for attack rates among subjects in the different age ranges (Table 2).

During the study period, only two positive inmates (0.3%) were hospitalized for COVID-19. The first, aged 54 years, was unvaccinated, currently tracheotomized for a previous laryngeal carcinoma, and was hospitalized 4 days after the first positive test for SARS-CoV-2, for 12 days; the second, aged 39 years, was fully vaccinated, reporting a previous renal transplant and hepatitis B virus (HBV)-related chronic liver disease, and was hospitalized for pneumonia 20 days after the first positive test, for 8 days, during which he underwent therapy with monoclonal antibodies. None of the positive subjects died with COVID-19.

**Figure 2:**
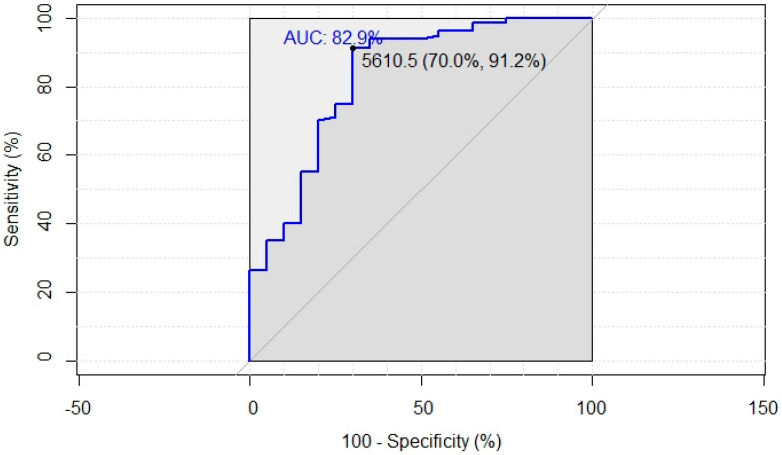
Receiver operating characteristic (ROC) analysis assessing the ability of anti-S IgG at T2 to predict neutralizing antibodies titer against wild type strain.

The group of 101 fully vaccinated healthy inmates investigated for the assessment of humoral immunity response showed a median age of 44.1 years (range 22–69 years). At T2, the median level of antibodies against S was 16322 ng/dL (range 1446–159,238 ng/dL). No significantly different neutralizing antibody titers between the Omicron and WT strains were found at T1 (5.0 vs. 6.1), while at T2 the titer was 10-fold lower against Omicron than against the WT strain (5.5 vs. 53.1, *p* < 0.001) (Table 3). ROC analysis for the WT strain showed that the area under the curve (AUC) was 0.829 (*p* < 0.001), with an optimal cut-off of 5610.5 ng/dL that maximized sensitivity (91.2%) and specificity (70.0%) (Figure 2). On the contrary, the analysis for the Omicron variant showed low power, considering the low number of inmates with neutralizing antibodies titers higher than 5.

## 4. Discussion

This study has described the features of a COVID-19 outbreak in a correctional facility during a period of high prevalence of the Omicron variant, according to the vaccination status of the prison population. Although possible immune escape from vaccine protection against Omicron variant infection has been described, our findings show that the booster dose of BNT162b2 mRNA vaccine was able to mitigate a COVID-19 outbreak in a congregate setting. Accordingly, the VN assay performed on serum samples of a subgroup of healthy inmates collected after the second dose of vaccine showed no production of neutralizing antibodies against Omicron in nearly all the subjects, supporting the need for a booster dose to prevent outbreaks in this particular high-risk setting.

In March 2021, about two months after the COVID-19 vaccines became available in Italy, by decree of the Italian Ministry of Health, prisoners as well as prison workers were included among the priority targets of the vaccination campaign due to the complexity of this population and their high risk of infection, severe disease, and death [19]. Unlike many other countries, this led to an immediate implementation of vaccinations within those categories in the Italian population [20]. At the time of the outbreak, the reported COVID-19 vaccination coverage in the investigated prisons showed 84.2% of inmates receiving at least one dose of mRNA vaccine, and 76.3% fully vaccinated, comparable to the coverage observed among Italian adults overall (83.1% and 76.3%, respectively), and the general Italian prison population at that time [21,22]. The high acceptance rate observed in the correctional setting under investigation is particularly relevant considering some concerns about high levels of vaccine hesitancy in prisons, including a survey showing a vaccination intention only in the 64.0% of 685 Italian detainees, lower than that expressed in the general population (84.1%) and in health care workers (80.7%) [23]. In this context, education and communication probably played an important role in mitigating refusals, avoiding vaccine hesitancy, and overcoming the lack of trust in medical services generally widespread in this population [13].

However, only 48.9%of the inmates under study received the booster dose, a percentage lower than the 55.1% reported in the Italian population. The administration of the booster dose among incarcerated people, therefore, seems to be challenging probably due to the high turnover of inmates. It is difficult to plan and complete the vaccination cycle six months after the second dose, with individuals often being released in the community or transferred to other prisons [24,25]. 

In January 2022, an outbreak of SARS-CoV-2 occurred in the investigated prisons, probably caused by the Omicron BA.1 variant, which showed an increase in its prevalence in Italy from 80% to 98% during the study period, replacing the delta variant [21].

Since the beginning of the pandemic, several reports have shown a higher risk of SARS-CoV-2 transmission in prisons and other congregate settings, with multiple large COVID-19 outbreaks in detention facilities worldwide, including before the Omicron spread [6,26,27]. We observed a global attack rate among inmates of 43.6%, higher than those reported during late 2020, before vaccination campaigns, from outbreaks within correctional facilities in England (17.9%) [28] and in Italy (19.3%) [29], but lower than those reported by other countries, such as the US (74.0%) [6]. These differences could be related to several factors, including the higher transmissibility of Omicron [30], but also the different implementation of containment measures according to prison configurations, the availability of staff, and the individual characteristics of prisoners (e.g., long-term or short-term sentence). Particularly, important differences in US health care, prison systems, and sociodemographic composition of the populations make it challenging to translate their findings into the European context.

According to vaccination status, the attack rate was higher in unvaccinated inmates (62.7%), while a significant lower percentage of positive subjects was detected among inmates with full vaccination (45.0%) and those with a booster dose (31.4%). These results seem to be the first evidence of the mitigating effect of mRNA vaccination, particularly the booster dose, on the spread of COVID-19 within correctional facilities during the Omicron wave, and are consistent with reports demonstrating the efficacy of vaccination against SARS-CoV-2 infection in congregate settings such as prisons [31,32]. Moreover, the observed protective effect of the booster dose is in line with previous reports indicating that the third dose of BNT162b2 boosts neutralization capability against Omicron to robust levels and may substantially reduce the risk of breakthrough infections [33,34]. Additionally, cases of severe COVID-19 or deaths were not detected in either the booster or non-booster cohorts, with only two subjects, one vaccinated and one unvaccinated, requiring hospitalization despite the large number of infections, confirming the milder effects of Omicron.

In the investigated outbreak, the attack rate was significantly lower in inmates who received their last vaccine dose 15 to 40 days before the positive test. According to this, Andrews et al. [35] reported that protection against infection is presumed to be highest in the first month after a booster dose. However, the neutralizing antibodies response and vaccine effectiveness decrease with increased time post-vaccination, and the long-term efficacy of a booster vaccination needs to be evaluated, especially considering that Omicron appears to accentuate the rapid waning of vaccine protection [36].

Although a definite immune correlate of protection against COVID-19 has not been established yet, neutralizing antibodies levels have been shown to correlate with vaccine efficacy in phase 3 studies across different vaccination platforms [37,38]. In line with previous studies, healthy inmates fully vaccinated with the BNT1622b vaccine achieved substantial detectable neutralizing titers against the WT strain [33,39,40]. These levels of neutralizing antibodies appeared to be slightly lower than those reported in previous studies using pseudotyped virus-based neutralization assays [33,41]. The observed differences could be related to the live-virus VN assay used in this study, which was found to be the most stringent among the VN assays, resulting in a lower GMT than other methods including plaque reduction, foci reduction, and pseudotyped virus-based neutralization assays [42]. The live-virus VN assay employed to evaluate neutralizing antibody titers in this population is a strength of this study, being the most specific and sensitive serological assay capable of evaluating and detecting functional neutralizing antibodies. However, GMTs found in our study were lower than those found in healthy subjects by a previous study using the same VN methodology, while they are similar to those observed in nursing home residents with a higher median age than our study population [43]. This evidence underlines a possible vulnerability of prison populations, even in the absence of chronic diseases, probably due to other concomitant conditions extremely common in this population, such as stress related to living in congregate settings, substance abuse, tobacco use, and alcohol abuse, that may have contributed to a lower immune response [44]. The relatively common immunosuppression and poor immune responses in the prison population underline the importance of vaccination in correctional settings, as a public health measure not only to protect high risk and marginalized subjects, but also to prevent the possible spread of new VOCs.

Remarkably, our results showed that Omicron neutralization was dramatically low after 21 days from the second mRNA vaccine dose, with a complete lack of neutralization in 90% of individuals and a GMT 10-fold lower than WT GMT, confirming previous reports that describe a 20- to 40-fold reduction in neutralizing titers [33,34,39,45]. Even if T-cell immunity probably contributes to the protection induced by mRNA vaccination, the poor responses against Omicron observed in inmates highlight the potential of this variant to evade neutralizing immunity induced by the vaccine, suggesting that two doses of BNT162b2 may not be sufficient to protect against Omicron infection. This hypothesis is supported by the higher attack rates in subjects who did not receive a booster dose [46]. Moreover, poor vaccination responders were identified against WT and Omicron strains among healthy inmates, independently from age, emphasizing the heterogeneity of antibody responses to mRNA vaccination in the general population [47]. As mRNA vaccination has only recently been implemented in large populations, the immunological basis of this heterogeneity is currently unknown. 

The findings in this report are subject to some limitations. First, it was not possible to evaluate attack rates among prison workers, because screening testing was not expected for them, limiting the ability to confirm the total number of COVID-19 cases in the prison considered as a unique setting. Second, it was not possible to perform a follow-up to evaluate the neutralizing antibodies response after the booster dose in the inmates evaluated after two doses. Moreover, it was not possible to evaluate certain potential factors interfering with the development of neutralizing antibodies, such as a tobacco habit or alcohol abuse, nor factors such as subjects’ compliance with preventive measures put in place, such as the use of masks in facilities, which could have had an impact on the transmission rate of COVID-19. Finally, although a very high prevalence of Omicron was reported during the study period, no specific analysis was performed on the VOC responsible for the outbreak in the correctional facility.

However, this is one of the first studies to evaluate and describe the COVID-19 outbreak attack rate according to vaccination status in a correctional setting during the Omicron wave. Moreover, the live-virus VN assay used to evaluate neutralizing antibody titers in this particular population is a strength of this study, being the most specific and sensitive serological assay capable of evaluating and detecting functional neutralizing antibodies.

## 5. Conclusions

In conclusion, the findings taken together support the need for an urgent widespread deployment of additional mRNA vaccine booster doses, as an essential public health measure to limit Omicron transmission and increase the protection of inmates and prison workers against COVID-19, in coordination with other prevention strategies to be maintained in correctional settings. Our results clearly indicate that refusing to prioritize incarcerated persons for vaccination should be considered irrational and unethical, suggesting a change of guidelines and policy for countries that omit plans for incarcerated people and prioritize only prison staff for vaccination [48,49]. Moreover, high rates of vaccine hesitancy observed among incarcerated people can be effectively countered by educational and communication campaigns about COVID-19 vaccines to be offered in correctional facilities. Nevertheless, while vaccination programs are crucial in reducing transmission among incarcerated people and to external communities, substantial risks may continue to exist in the penitentiary system, as prison conditions continue to be overcrowded and unsanitary, and the effects of variant strains are yet unknown [50].

## Figures and Tables

**Table 1:** Age, days from the last dose of vaccine, and vaccination status among the inmates participating to the screening campaign during the study period.

	Inmates (N. 595)				
Characteristics	Unvaccinated (15.8%)	Partially Vaccinated (7.9%)	Fully Vaccinated (27.4%)	Boostered (48.9%)	Total (100.0%)
Age groups (years) ^a^					
18–29	4.1	1.6	5.5	6.1	17.3
30–39	5.1	1.2	10.7	13.0	30.0
40–49	4.3	3.1	5.5	13.3	26.2
50–59	1.0	1.5	3.9	9.5	16.1
≥60	1.3	0.5	1.6	7.0	10.4
Days since the last vaccine dose ^a^					
≤14	-	0.8	2.0	13.2	16.0
15–40	-	3.6	4.5	25.1	33.4
41–89	-	0.8	8.7	8.7	18.3
≥90	-	2.6	12.2	1.6	16

^a^ *p* < 0.001.

**Table 2:** SARS-CoV-2 attack rates and relative risk (RR) among inmates in the three prisons under study, by vaccination status, days since the last dose of vaccine, and age.

Characteristics	No. of Cases (Population)	Attack Rate %	RR (CI 95%)
Vaccination status *			
-Unvaccinated ^a^	59 (94)	62.7	1.8 (1.5–2.3)
Partially vaccinated	23 (42)	52.3	1.5 (1.0–1.9)
Fully vaccinated	68 (151)	45.0	1.3 (1.0–1.6)
Boostered ^a^	68 (213)	31.4	0.7 (0.6–0.9)
Total	218 (500)	43.6	0.8 (0.2–3.0)
Days since the last vaccine dose			
15–40 ^a^	57 (199)	28.6	0.6 (0.5–0.8)
41–90	50 (109)	45.8	1.2 (1.0–1.6)
>90	52 (99)	52.5	1.5 (1.2–1.8)
Age groups (years)			
18–29	40 (103)	38.9	1.0 (0.7–1.3)
30–39	74 (178)	41.5	1.1 (0.9–1.4)
40–49	62 (156)	39.7	1.1 (0.8–1.3)
50–59	27 (96)	28.0	0.7 (0.5–0.9)
≥60	22 (62)	35.4	0.9 (0.6–1.3)

^a^ *p* < 0.001; * excluding inmates receiving the last dose in the 14 days before the testing.

**Table 3:** Neutralizing antibodies geometric mean titers against the wild-type strain and the Omicron variant, 21 days after the first (T1) and 21 days after the second mRNA vaccine dose (T2), in a sub-group of healthy inmates.

Neutralizing Antibodies	T1 (N.101) GMT	T2 (N.101) GMT ^a^
Anti-wild type	6.1	53.1
Anti-Omicron	5.0	5.5

^a^ *p* < 0.001.

## Data Availability

Not applicable.
